# Supplementary material for: CD112 Regulates Angiogenesis and T Cell Entry into the Spleen
Source: Cells. 2021 Jan 15;10(1):169. doi: 10.3390/cells10010169 (PMC7830896; doi:10.3390/cells10010169)
Supplement: Supplementary file 1 [file cells-10-00169-s001.pdf]

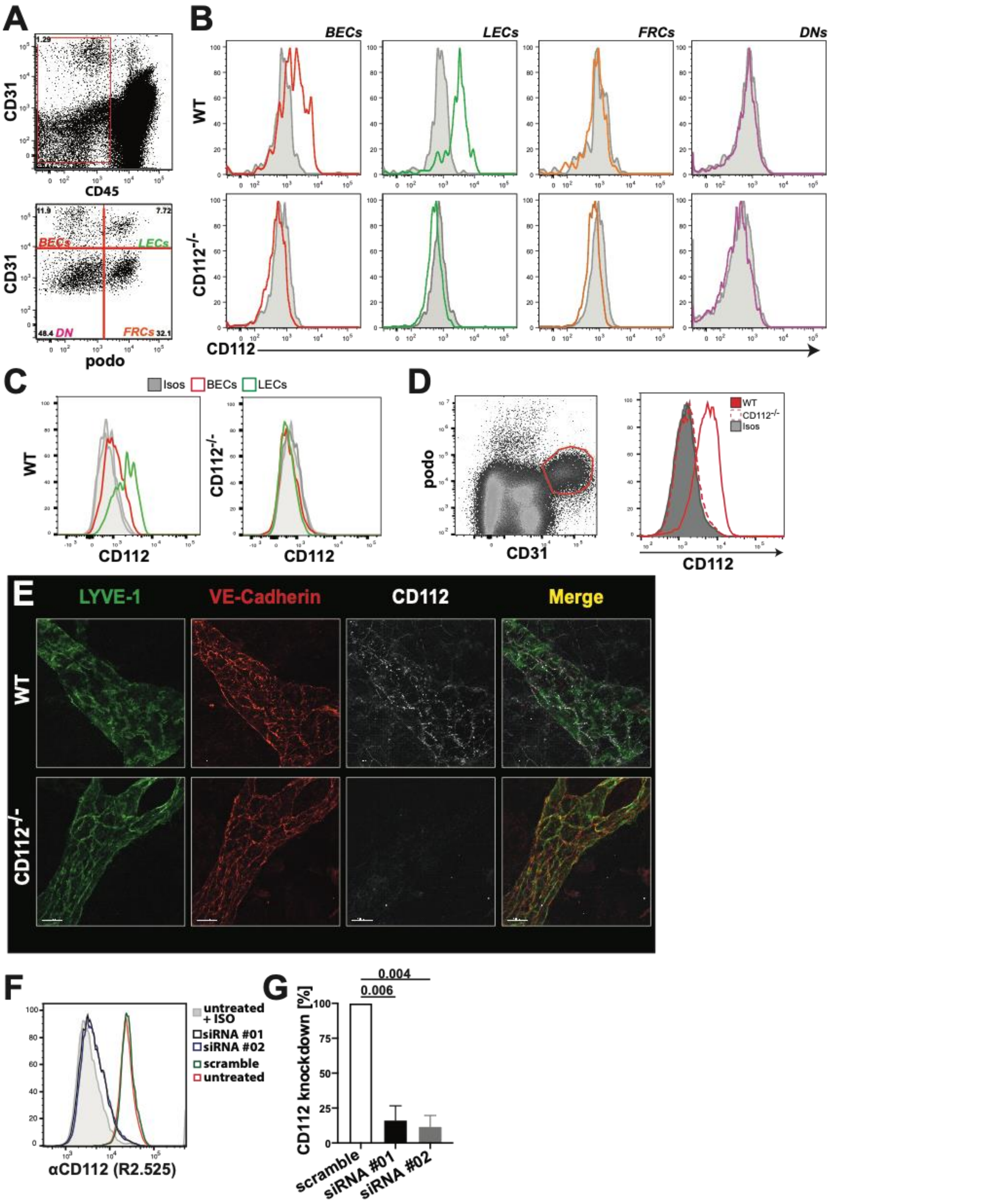

Supplemental Figure S1: CD112 is expressed in lymph nodes and diaphragm (**A,B**) Single cell suspensions of popliteal and cervical LNs of CD112$^{-/-}$ mice and littermate controls were analysed by flow cytometry. (**A**) Gating scheme of LN stromal cells: FRCs (CD31^{-}podo^{+}), DN (double negative) (CD31^{-}podo^{-}), BECs (CD31^{+}podo^{-}) and LECs (CD31^{+}podo^{+}). (**B**) Expression of CD112 in the different stromal cells isolated from LNs from WT and CD112$^{-/-}$ mice. Representative plots from one experiment are shown. (**C**) Flow cytometry analysis of ear skin in CD112$^{-/-}$ mice in LECs (CD31^{+}podo^{+}) and BECs (CD31^{+}podo^{-}). Representative histograms from two independent experiments are shown. (**D**) Analysis of CD112 expression murine spleen of CD112$^{-/-}$ and WT mice. BECs were identified as

$CD31^+podo^-$ cells (left). CD112 expression in $CD112^{-/-}$ splenic BECs (right). Representative plots are shown from three independent experiments. (**E**) Immunofluorescence staining performed on diaphragm from WT and $CD112^{-/-}$ mice further showing expression of CD112 on lymphatic and blood vasculature. Scale bar: 40 μm. Data from one experiment are shown. (**F,G**) CD112 binding by antibody R2.525 is lost upon siRNA-mediated knockdown of CD112 in human LECs. Human LECs were treated with either scramble or CD112-specific siRNA (siRNA#01 and siRNA#02) and subjected to flow cytometry analysis. (**F**) Representative histogram from one out of three independent experiments is shown. Grey: untreated + isotype control, red: untreated + R2.525, green: scramble siRNA + R2.525, black: siRNA#01+ R2.525, blue: siRNA#02 + R2.525. (**G**) Quantification of the knockdown efficiency observed in three different experiments: Untreated + isotype MFI values were subtracted from all CD112 (+ R2.525) MFI values and normalized to the scramble MFI value. Pooled data from three independent experiments.

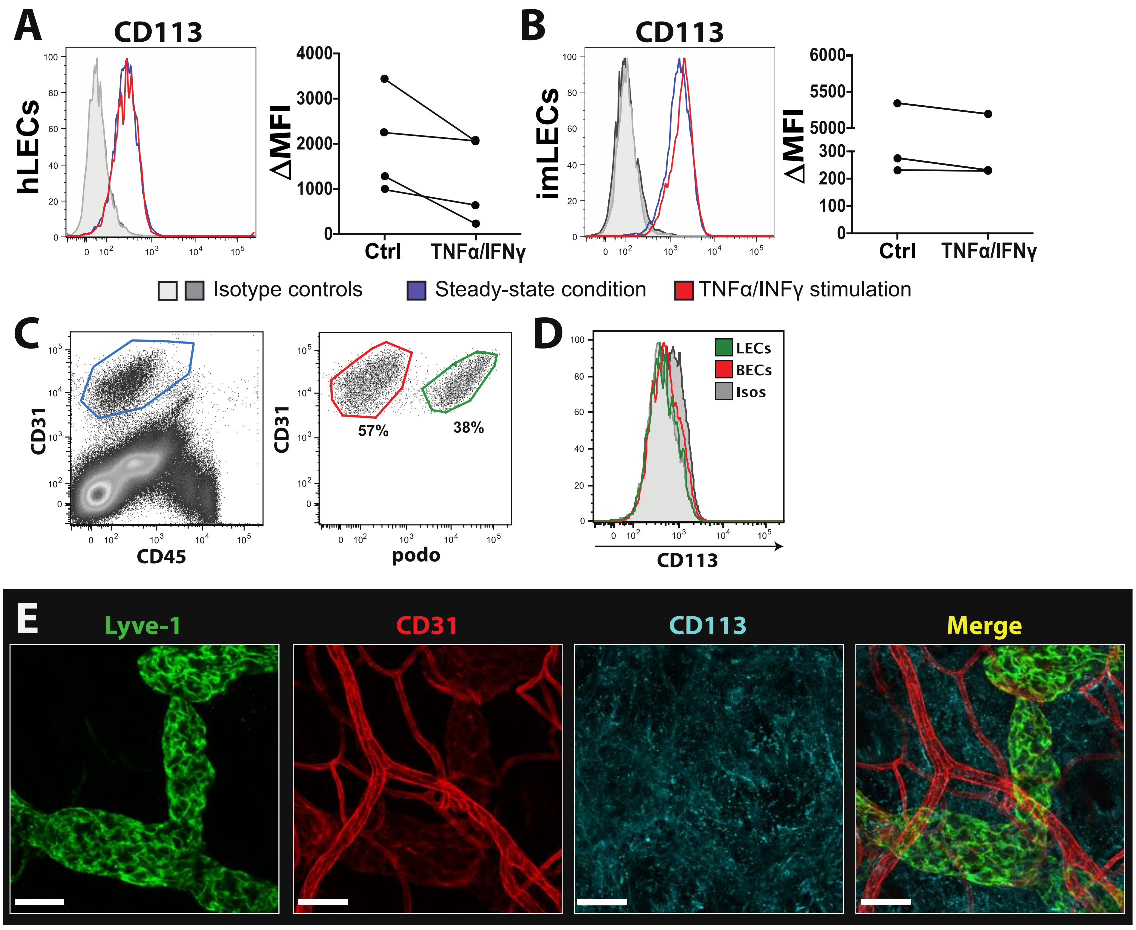

Supplemental Figure S2: CD113 is not expressed by endothelial cells in vivo. Flow cytometry analysis confirming CD113 expression by human (**A**) and conditionally immortalized murine LECs (**B**). Representative histograms of CD113 expression comparing steady-state (blue line) and inflamed condition (red line: TNFα/IFNγ treated; grey lines: isotype controls) (left). Summary of MFI values of CD113 expression of three to four experiments are shown on the right. Data points of the same experiment are connected by a line. (**C, D**) Flow cytometry analysis of CD113 expression in mouse ear skin single cell suspensions. (**C**) Endothelial cells were identified as $CD31^+CD45^-$ cells (left) and further divided into BECs and LECs based on podoplanin (podo) expression (right). (**D**) Representative histogram of CD113 expression in LECs (Green; $CD31^+podo^+$) and BECs (red; $CD31^+podo^-$). (**E**) Confocal images of ear skin whole-mount immunofluorescence staining of CD113 expression (blue) by lymphatic and blood vessels. Scale bar: 50 μm.

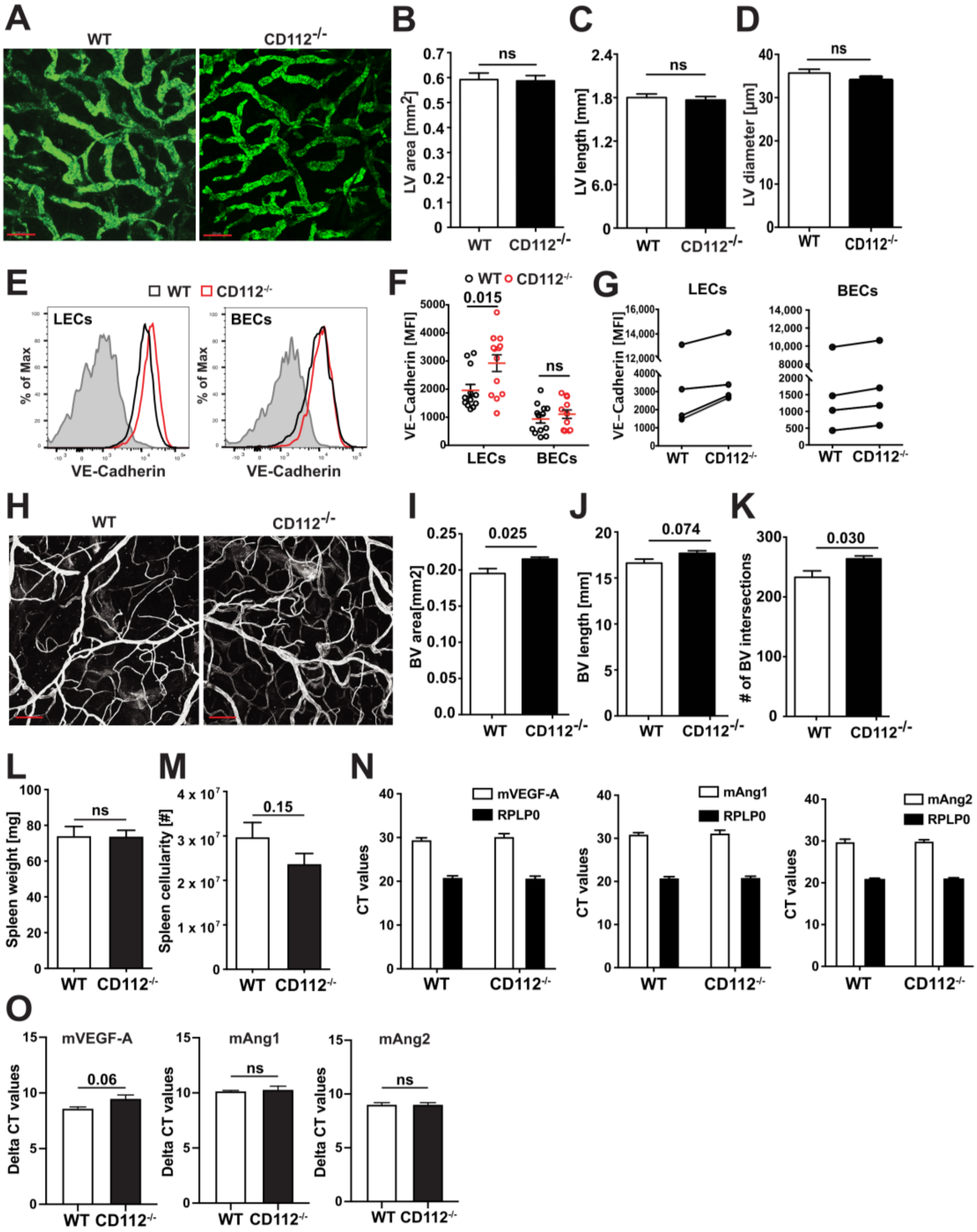

Supplemental Figure S3: CD112$^{-/-}$ mice display no aberrant lymphatic vessel phenotype. (**A–D**) The LYVE-1^{+} lymphatic vasculature in ear skin of 7-week-old WT and CD112$^{-/-}$ mice was analysed by immunofluorescence whole mount staining. (**A**) Representative images of ear skin whole mount stained for LYVE-1^{+} lymphatic vessels. Scale bar: 200 µm. (**B–D**) Parameters analysed by morphometric image analysis include (**B**) area, (**C**) lymphatic vessel length, (**D**) diameter calculated through the ratio between area and length. Pooled data obtained from 3–4 mice/group are shown. (**E,F**) Flow cytometry analysis of VE-cadherin expression in ear skin LECs in CD112$^{-/-}$ mice. (**E**) Representative histograms of VE-cadherin expression in LECs and BECs single cell suspension generated from mouse ear skin (black line: WT, red line: CD112$^{-/-}$, grey: isotype control). (**F**) Pooled MFI values from three independent experiments. Each dot represents a mouse (n = 13 mice/group). (**G**) Summary of the mean MFI values of VE-cadherin expression measured in ear skin LECs (left) and BECs (right) in four independent experiments (n

= 3–4 mice/group/experiment). Values from the same experiment are connected by a line. (**H–K**) MECA-32$^+$ blood vasculature in ear skin WT and CD112$^{-/-}$ mice was analysed by immunofluorescence whole-mount staining. (**H**) Representative images of ear skin whole mount stained for MECA-32$^+$ blood vessels. Scale bar: 200 μm. (**I–K**) Analysis of blood vessel morphology parameters such as (**I**) area, (**J**) blood vessel length and (**K**) intersections. Data from 5 mice/group from one experiment are shown. (**L,M**) Cellular analysis of WT and CD112$^{-/-}$ spleens. Pooled data of spleen weight (n=11 mice/group) (**L**) and spleen cellularity (**M**) (*n* = 16–17 mice/group), ns = not-significant. (**N,O**) Quantitative real-time PCR analysis showing mRNA levels of mouse VEGF-A, angiopoetin-1 (mAng1) and angiopoetin-2 (mAng2) in whole spleen tissue lysates of WT and CD112$^{-/-}$ mice. RPLP0 served as an internal control. The experiment was performed in triplicates. (**N**) Averaged threshold Cycle (CT) values per mouse for each gene. (**O**) Delta CT values normalized to RPLP0 (*n* = 3 mice/group).

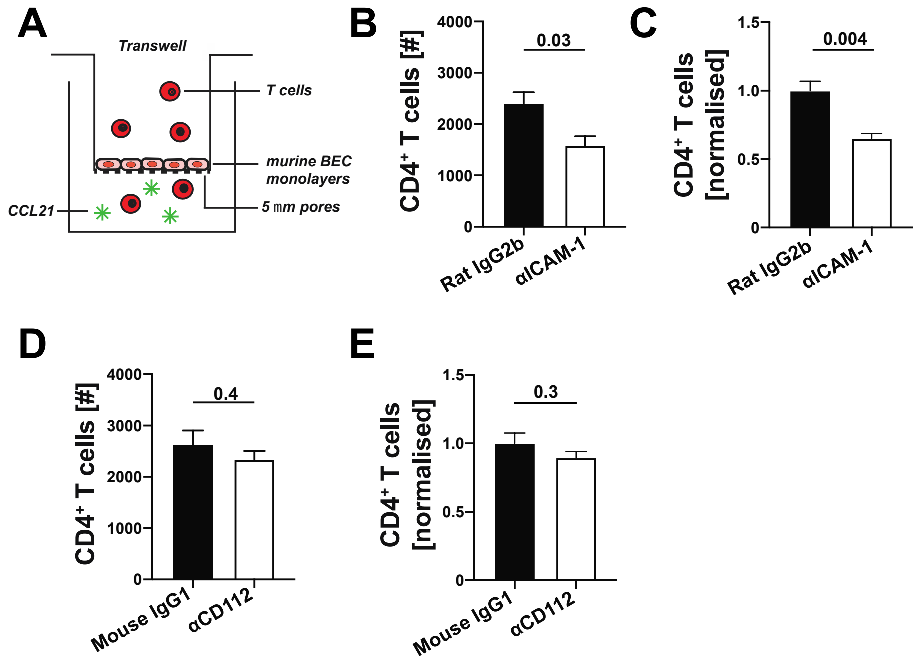

Supplemental Figure S4: CD112 blockade does not reduce TH1 transmigration through BECs in vitro. (**A**) Schematic representation of an in vitro transendothelial cell migration experiment, in which T cells were added in the top chamber and CCL21-induced transmigration through an endothelial cell monolayer (MS-1) was quantified in the bottom chamber by flow cytometry after 4 hours. (**B–E**) Transmigration of *in vitro* generated TH1 $CD4^{+}$ T cells across MS-1 monolayer was investigated upon either (**B,C**) ICAM-1 blockade (clone YN1, rat IgG2b) or (**D,E**) CD112 antibody blockade (clone R2.525, mouse IgG1). (**B,D**) show absolute numbers of transmigrated cells, (**C,E**) show values normalized to the isotype control. Pooled data from two independent experiments are shown (n = 3 replicates per group and per experiment, i.e. 6 per pooled group).

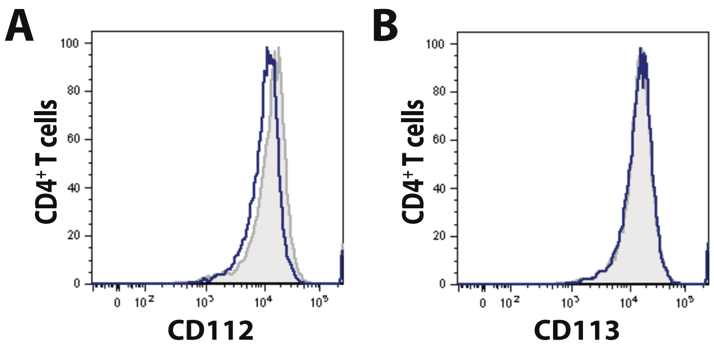

Supplemental Figure S5: Murine T cells do not express CD112 or CD113. (**A,B**) Flow cytometry analysis of isolated murine CD4+ T cells. Representative histograms of CD112 (**A**) and CD113 (**B**) expression in murine CD4+ T cells are shown from one out of three similar experiments.

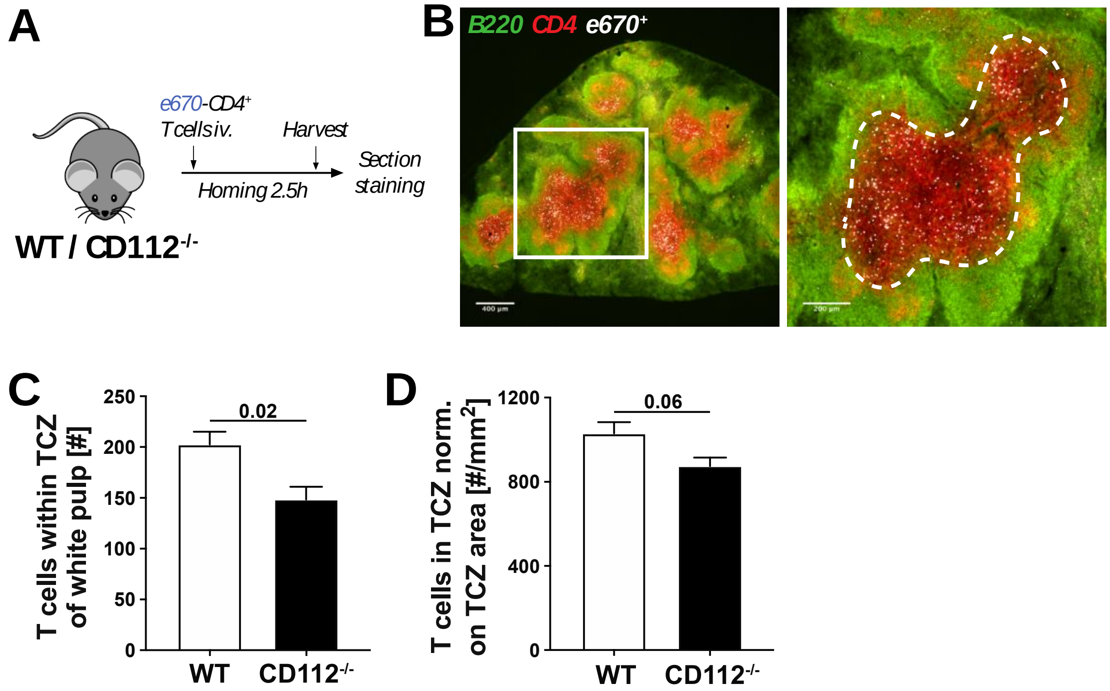

Supplemental Figure S6: T cells home less efficiently into the spleen of CD112$^{-/-}$ mice. (**A–D**) T cell homing experiment to the spleen analysed with immunofluorescence. (**A**) Freshly isolated CD4^{+} T cells were fluorescently labelled with eFluor670 (eBioscience) and injected intravenously into WT or CD112$^{-/-}$ mice. After 2.5 hours, spleens were harvested and embedded in optimal cutting temperature (OCT) compound and 50 µm sections were prepared. (**B**) Sections were stained for B cell marker B220 (green) and CD4 (red). Overview image of a WT spleen stained for B220 and CD4 (left). Scale bar: 400 µm. Confocal images of T cell zones (red) within splenic B cell follicles (red). Scale bar: 200 µm. Adoptively transferred e670^{+} T cells (white dots) were enumerate within the T cell zone (dotted white line) of the B cell follicles using the particle analyser in FIJI (ImageJ). (**C,D**) Analysis of the absolute numbers (**C**) or numbers normalized on the area of the T cell zone (**D**) of T cells homed into the spleens of WT and CD112-deficient mice. One out of three independent experiments is shown (4–6 mice/group). 5–10 images/mouse/experiment were analysed.
